# Monitoring of telomere dynamics in peripheral blood leukocytes in relation to colorectal cancer patients’ outcomes

**DOI:** 10.3389/fonc.2022.962929

**Published:** 2022-09-20

**Authors:** Kristyna Tomasova, Michal Kroupa, Alzbeta Zinkova, Marie Korabecna, Veronika Vymetalkova, Pavel Skrobanek, Ladislav Sojka, Miroslav Levy, Kari Hemminki, Vaclav Liska, Petr Hosek, Rajiv Kumar, Ludmila Vodickova, Pavel Vodicka

**Affiliations:** ^1^ Department of the Molecular Biology of Cancer, Institute of Experimental Medicine of the Czech Academy of Sciences, Prague, Czechia; ^2^ Biomedical Center, Faculty of Medicine in Pilsen, Charles University, Pilsen, Czechia; ^3^ Institute of Biology and Medical Genetics, First Faculty of Medicine, Charles University, Prague, Czechia; ^4^ Department of Oncology, First Faculty of Medicine, Charles University and Thomayer Hospital, Prague, Czechia; ^5^ Department of Surgery, First Faculty of Medicine, Charles Univesity and Thomayer Hospital, Prague, Czechia; ^6^ Division of Cancer Epidemiology, German Cancer Research Center, Heidelberg, Germany; ^7^ Department of Surgery, Medical Faculty in Pilsen, Charles University, Pilsen, Czechia; ^8^ German Cancer Research Center, Heidelberg, Germany

**Keywords:** leukocyte telomere length, colorectal cancer, *TERT* expression, adjuvant therapy, longitudinal monitoring

## Abstract

We investigated the possible associations between leukocyte telomere length, therapy outcomes, and clinicopathological features in patients with colorectal cancer. Additionally, telomerase reverse transcriptase (*TERT*) expression was evaluated. Telomere length was measured using singleplex qPCR in 478 consecutive leukocyte DNA samples from 198 patients. Blood was drawn at diagnosis prior to any therapy and then at 6-month intervals for 18 months. Following diagnosis, the telomeres gradually shortened during the course of the treatment regardless of the patient’s age. The most pronounced decrease was observed 12 months after the diagnosis (p < 0.0001). Based on tumor localization, the decrease in telomere length one year after the diagnosis followed different trajectories (p = 0.03). In patients treated with adjuvant therapy, telomere length correlated with the time elapsed after completion of therapy (p = 0.03). *TERT* expression did not correlate with the telomere length; however, it was higher in women than men (1.35-fold, 95% CI 1.11–1.65, p = 0.003) and in smokers than non-smokers (1.27-fold, 95% CI 1.01–1.61, p = 0.04). Leukocyte telomere length declines naturally during aging, but the accelerated shortening observed in our patients was age-independent. Telomere length manifestly reflected chemotherapy impact and could be linked to therapy toxicity.

## 1 Introduction

Telomeres, which are protective caps at the chromosomal ends, comprise hexanucleotide tandem repeats in combination with the associated shelterin complex subunits. Telomere complexes protect chromosomal ends from DNA double-strand break repair machinery, which can otherwise initiate inappropriate recombination events leading to extensive chromosomal rearrangements (1). Telomeres, however, due to the intrinsic limitations of DNA replication undergo gradual age-dependent attritions (2, 3). Telomere function is also affected by oxidative DNA damage caused by endogenous and exogenous agents (1, 2). Excessive telomere shortening can trigger irreversible cell proliferation arrest, leading to the accumulation of senescent cells and aging. Cancers develop mechanisms to bypass the replicative senescence and become immortal through upregulation of telomerase reverse transcriptase (*TERT*), which is a subunit of the holoenzyme that elongates telomeres (4).

Anticancer drugs perturb telomere length, either by their direct DNA-damaging activity or secondary-induced oxidative stress. Their effect depends on the treatment regimens and cancer types (5–7). Currently, only a limited number of studies have focused on the effect on telomere length dynamics during the therapy in solid tumors (5). Benitez-Buelga et al. observed a negative correlation between mean leukocyte telomere length (LTL) and the duration of cancer therapy in 489 patients with sporadic breast cancer (7). The shortening effect was significant in patients treated for > 90 days, and their LTL remained shortened until one year after the end of the treatment. However, other longitudinal studies on patients with solid tumors have not reported any association between therapy received and LTL (8, 9). To the best of our knowledge, only one study has investigated the therapeutic effect on LTL in colorectal cancer (CRC) patients, featuring, however, a very small (n = 32) and diverse cohort of solid cancer patients, including only three CRC patients (10).

Upregulation of telomerase has been reported in hematopoietic cells following consecutive chemotherapy courses and is interpreted as a mechanism to compensate for telomere attrition (11). A study of childhood cancers by Franco et al. (12) reported that telomerase activity does not change in any phase of treatment in solid tumors, but rather decreases sharply during the induction chemotherapy in acute lymphoblastic leukemia due to the elimination of the leukemic clone (12).

Epidemiological studies have documented a complex association between disrupted LTL homeostasis (either extremely short or long LTL phenotypes) and different cancers (2, 13). It has resulted in an intensive investigation of LTL as a risk and prognostic marker for patients with cancer. Specifically, in CRC, there is a need for new biomarkers to predict therapeutic response, toxicity, and resistance. However, the question remains whether a variation in *TERT* expression and LTL, possibly affected by DNA-damaging anticancer drugs, can be the biological mechanism underlying the inadequate response to cancer therapy. Moreover, in CRC patients, the effect of treatment on the constitutive telomere length changes and *TERT* expression has not yet been investigated. Our study hypothesizes that LTL and *TERT* expression are surrogate markers of cancer therapy sensitivity, toxicity, and overall patient outcomes.

## 2 Material and methods

### 2.1 Population characteristics

One hundred and ninety-eight newly diagnosed and histologically confirmed patients diagnosed with CRC (median age 66 years; range 32–88 years; 127 men; 71 women) were enrolled in the study. Peripheral blood samples were collected from all patients at Thomayer University Hospital in Prague, the Czech Republic, from June 2008 to May 2018. In total, 478 samples were collected from 198 patients. The characteristics of the study group are shown in **Table 1**. Blood specimens were drawn from patients at the baseline before any treatment (n = 137), followed by collection at the sixth (n = 156), twelfth (n = 135), and eighteenth (n = 50) month after diagnosis. The 6-month sampling encompassed, for the most part, patients (56%) for whom the anticancer therapy had been completed. An age-matched control group was not included, based on our previous results confirming that the lymphocyte telomere length of cancer patients is age-independent, in contrast to healthy controls (14).

**Table 1:** Patients’ characteristics.

Studied cohort of patients	median age (years) [range]	66 [32-88] n = 198	%
**Gender**			
	males	127	64.1
	female	71	35.9
**Smoking status**			
	smokers	46	24.5
	non-smokers	142	75.5
**Tumor site location** ^a^			
	proximal colon	35	18.0
	distal colon	86	44.3
	rectum	73	37.6
**UICC TNM stage** ^b^			
	I + II	118	63.8
	III + IV	67	36.2
**Microsatellite status**			
	stable	138	85.2
	unstable	24	14.8
**Therapy response**			
	good	64	70.3
	poor	27	29.7
**Neoadjuvant therapy**			
	yes	66	33.3
	no	132	66.6
**Adjuvant therapy**			
	yes	89	46.6
	no	102	53.4

a Patients with CRC were categorized as having proximal colon (C18.0-18.4), distal colon (C18.5.-19.0), or rectal cancer (C20) according to the International Statistical Classification of Diseases and Related Health Problems 10^th^ Revision (ICD-10).

b Tumor-Node-Metastasis (TNM) staging system was developed by the Union for International Cancer Control (UICC) to classify the anatomical extent of tumor cancers.

The table summarizes the characteristics of all patients who participated in the study. Number of patients do not always add up to 100% (n = 198) due to missing data for some attributes.

In our study, we analyzed the effect of both neoadjuvant and adjuvant therapies on LTL. In general, for CRC, the type of treatment varies according to the tumor localization. The treatment strategy for colon cancer patients typically involves surgical removal, followed by adjuvant chemotherapy based on 5-fluorouracil (often in combination with oxaliplatin, irinotecan, or either of the above drugs with added folic acid (15). In contrast, rectal cancer treatment is based on neoadjuvant (5-fluorouracil-based chemo)radiotherapy to downstage the tumor before surgical removal. Patients with locally advanced tumors may sometimes receive adjuvant chemotherapy (based on drugs described above for colon cancer treatment). In the study, we also stratified patients based on their response to the therapy. Good therapy responders were defined as patients who did not require a switch of chemotherapy regimen, achieved remission after a prescribed number of cycles, and did not experience any chemotherapy side effects.

The cohort analyzed for the impact of 5-fluorouracil-based chemotherapy as standard care for CRC in the adjuvant setting comprised 42 individuals with a complete record of a given treatment. To minimize the influence of other treatment strategies, only patients without previous neoadjuvant treatment were enrolled. The effect of neoadjuvant therapy was investigated in 23 patients with rectal cancer with complete treatment records. To eliminate the possible influence of other administered treatments, the cohort included only patients who did not receive postoperative therapy. Neoadjuvant therapy for the patients involved (5-fluorouracil-based chemo)radiotherapy or chemotherapy with 5-fluorouracil used alone. The therapies prescribed for each patient are presented in **Supplementary Table 1**.

All participants provided written informed consent to participate in this study. Peripheral blood sampling was performed in accordance with the ethical standards outlined in the Declaration of Helsinki. The clinicopathological characteristics of all patients were collected from their medical records. Personal data were acquired using lifestyle questionnaires. Only collaborating clinicians could disclose participants’ identities since they entered the anonymized study.

### 2.2 Peripheral blood collection, processing, and DNA and RNA extraction

Blood was collected into Vacuette K3EDTA blood collection tubes by venipuncture. After sampling, fresh specimens were processed to produce mononuclear cells. These peripheral mononuclear cells were isolated from 2 ml of whole blood by Ficoll-Paque PLUS (GE Healthcare Life Sciences) flotation, as recommended in the manufacturer’s handbook, and frozen in TRI Reagent (Sigma-Aldrich). The remaining whole blood was stored at -80°C.

Genomic DNA from whole blood (i.e., leukocytes) and total RNA from mononuclear cells were used as starting materials for qPCR-based analyses. DNA was extracted from 200 µL of blood according to the DNeasy Blood and Tissue Kit protocol (Qiagen, Valencia, CA). RNA was extracted from Ficoll-isolated mononuclear cells using the standard TRI Reagent protocol.

DNA and RNA concentrations were determined using a NanoDrop 2000 spectrophotometer (Thermo Fisher Scientific). RNA quality (RIN ≥ 8) was assessed using a Bioanalyzer 2100 (Agilent, Santa Clara, CA, USA) and the Agilent RNA 6000 Nano kit.

### 2.3 Measurement and calculation of telomere length

Relative LTL was estimated using singleplex qPCR based on Cawthon (16) and later optimized by Gil and Coetzer (17). The method was adopted in collaboration with the Department of Biology and Medical Genetics, First Faculty of Medicine, Charles University, Prague, which has published articles on this topic (18, 19).

Briefly, LTL measurements were performed using the ViiA 7 Real-Time PCR System (Applied Biosystems, Foster City, CA, USA). qPCR reactions were run on a MicroAmp Fast Optical 96-Well Reaction Plate (Life Technologies, Carlsbad, CA, USA) in triplicate. The 24 µL reactions consisted of PowerUp SYBR Green Master Mix (Life Technologies, Carlsbad, CA, USA), primers (300 nM for *36B4*, 900 nM for telomeres), and 5 ng of DNA and were prepared according to the manufacturer’s instructions. To detect the reference *36B4* gene, which encodes the acidic ribosomal phosphoprotein P0, and telomere sequences, primers (Sigma-Aldrich, St. Louis, MO, USA) with the same annealing temperature were used. Primer sequences are listed in **Supplementary Table 2**. The reaction conditions were identical for both targets. The holding stage was performed at 50°C for 2 min and 95°C for 2 min, followed by 40 cycles at 95 °C for 30 s and 54°C for 1 min (ramp rate: 1.6°C/s). Primers’ specificity was assessed by melting curve analysis (**Supplementary Figure 1**) at the end of the amplification procedure. A calibration curve and interplate calibration were established using a 17 ng/µL reference DNA sample pooled from 30 healthy middle-aged hospital volunteers: 15 females and 15 males ranging from 41 to 55 years of age with no previous experience with cancer (20). Participants were recruited at the Faculty Hospital Kralovské Vinohrady in Prague, Czech Republic. Amplification efficiency was verified using a 5-point calibration curve created through a 2-fold serial dilution of the reference sample with three replicates at each point. The DNA amount ranged between 17 ng to 1.0625 ng per well. The reaction efficiency was calculated from the slope of the standard curves (see **Supplementary Figure 2**) using the equation: E = -1 + 10^(-1/slope)^ and evaluated using Viia7 software. The efficiency was 97% for telomeres and 101% for *36B4*. The interplate and intraplate coefficients of variation were 1.03% and 0.35% for *36B4* and 1.92% and 0.24% for telomeres, respectively. Samples assayed in triplicate with the standard deviation (SD) > 0.3 were omitted from the analysis, and their measurement was performed again. When two replicates were nearly identical, and one was distant with a suspected dilution error, the outlier was omitted from the calculations.

LTL was estimated as the ratio of telomere repeat (T) copy number to single gene (S) copy number (T/S ratio) and calculated by the ΔΔCt method (21). The mean cycle threshold (Ct) of *36B4* was subtracted from the mean Ct of telomeres. The calculated ΔCt of each sample and ΔCt of the standard (derived from the ΔCts of standard replicates within the calibration curve) were substituted into the formula 2^-(ΔCt(sample)-ΔCt(standard)^. The obtained T/S ratio is proportional to the average TL in the cell. A ratio above 1 indicated that the patient had longer telomeres than the reference sample.

### 2.4 Measurement and calculation of *TERT* mRNA expression

*TERT* mRNA expression levels were measured by two-step singleplex quantitative reverse-transcription PCR (RT-qPCR) using the TaqMan chemistry.

Complementary DNA (cDNA) was synthesized using 300 ng of total RNA with a High Capacity cDNA Reverse Transcription Kit (Life Technologies, Carlsbad, CA, USA) in 15 μL reactions. All components were combined and incubated in an MJ Research PTC-200 Thermal Cycler (Bio-Rad), following the manufacturer’s instructions. Subsequent qPCR reactions were carried out in a 384-well format using a MicroAmp Optical 384-Well Reaction plate (Life Technologies, Carlsbad, CA, USA) on the Viia7 Real-Time PCR System (Applied Biosystems). All reactions were performed in triplicates. To normalize *TERT* expression levels, endogenous control genes glyceraldehyde-3-phosphate dehydrogenase (*GAPDH*) and beta-actin (*ACTB*), which are known to be stably expressed in mononuclear cells (22), were used. All 20 μl qPCR reactions included TaqMan Universal Master Mix II, with UNG (Applied Biosystems, Carlsbad, CA, USA), particular FAM/MGB TaqMan Gene Expression Assay (Hs00972650_m1 (TERT), Hs01060665_g1 (ACTB), or Hs02786624_g1 (GAPDH)), RNase-free water, and 2 μl of 3:1 diluted cDNA. Thermocycling conditions were 50°C for 2 min and 95°C for 10 min, followed by 40 cycles at 95°C for 15 s and 60°C for 1 min. The fluorescence signal was captured at the end of the 60 °C segments. Using the peripheral blood mononuclear cell RNA from a healthy donor, the interplate and intraplate coefficients of variation were 2.21% and 0.45% for *TERT*, 0.70% and 0.42% for *GAPDH*, and 0.40% and 0.49% for *ACTB*. Technical replicates with the SD of the average Ct > 0.3 were omitted from the study, and samples were measured in a new run.

The relative expression of *TERT* was calculated analogically to the previous description (see **Section 2.3**) using the mean Ct of the two housekeeping genes as a reference.

### 2.5 Statistical methods

Statistical analysis was performed using STATISTICA (version 11Cz; TIBCO Software Inc., Palo Alto, CA, USA) and MATLAB (version 2019b; The MathWorks, Inc., Natick, MA, USA). All reported p-values were two-tailed and the level of statistical significance was set at α = 0.05.

Owing to their mostly normal distribution, LTL and log_2_-transformed *TERT* expression values (i.e., -ΔΔCt values) were compared between relevant group pairs (according to sex, smoking habit, microsatellite instability, Union for International Cancer Control (UICC) tumor-node-metastasis (TNM) classification, and treatment response) using a two-sample *t*-test. The Mann-Whitney U test was used to compare the LTL of rectal cancer patients measured before and after neoadjuvant therapy (in an independent arrangement because of a small overlap between the patient groups). In the samples collected at the time of the diagnosis, an ordinal association between LTL and *TERT* expression was analyzed using the Kendall rank correlation coefficient. Kendall’s τ rank test was also performed to investigate the relationship between age and LTL or *TERT* expression and the correlation between LTL and time from adjuvant therapy termination. One-way analysis of variance (ANOVA) was used to examine the differences in LTL and *TERT* expression in patients diagnosed with proximal colon, distal colon, and rectal cancer. To examine LTL changes over time, these diagnosis-related groups were further investigated using a repeated-measures ANOVA. Determination of whether LTL values of all patients changed over time was also performed using repeated-measures ANOVA. Lastly, repeated-measures ANOVA was used to compare LTL in patients with different TNM stages and treatment responses over time. Kruskal-Wallis ANOVA was used to compare the mean LTL values in time intervals from adjuvant therapy. Subsequently, to verify the time-related LTL changes in the context of other variables and account for possible confounding, while also allowing for patients with incomplete data to remain in the analysis (as opposed to repeated-measures ANOVA), a linear mixed-effects model was fit in the data, including the sampling time as a continuous predictor, TNM category, primary tumor location (colon/rectum), radiotherapy (yes/no), and application of 5-fluorouracil (yes/no) as fixed categorical factors, and the patient ID as a random factor.

Overall survival (defined from diagnosis to death or censored at the last follow-up) and its association with LTL were analyzed using the Kaplan-Meier method, Cox proportional-hazards model, and after stratification by LTL median, the Gehan-Wilcoxon test. The median follow-up time was 26.1 months (determined using the inverse Kaplan-Meier method).

## 3 Results

### 3.1 Leukocyte telomere length dynamics in a 12-month interval

**Figure 1:**
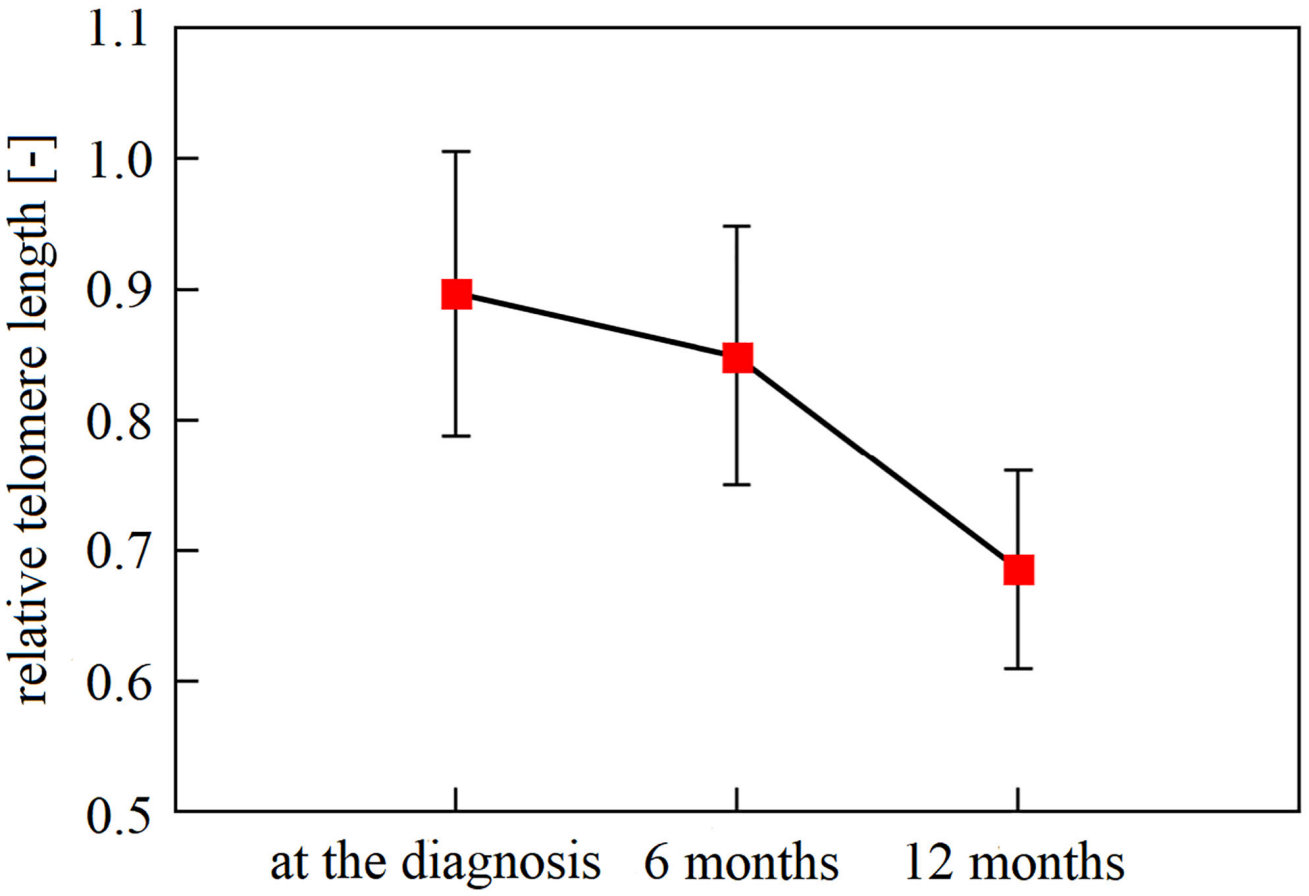
The downward trend in leukocyte telomere length of newly diagnosed patients within a 12-month interval. Mean LTL declined significantly over the recorded time points (p < 0.0001), with a gradual decline. With Bonferroni adjustment, patient leukocytes sampled at the time of diagnosis and in the following 6 months had longer telomeres than one year since diagnosis (n = 49, Bonferonni *post-hoc* test p ≤ 0.0001). Observation within 18 months could not be performed due to the paucity of samples taken 18 months after diagnosis (a complete number of blood samplings (at the diagnosis, 6 months, 12 months, and 18 months) has been accomplished only in 6 patients). Vertical bars denote 0.95% CI.

**Figure 2:**
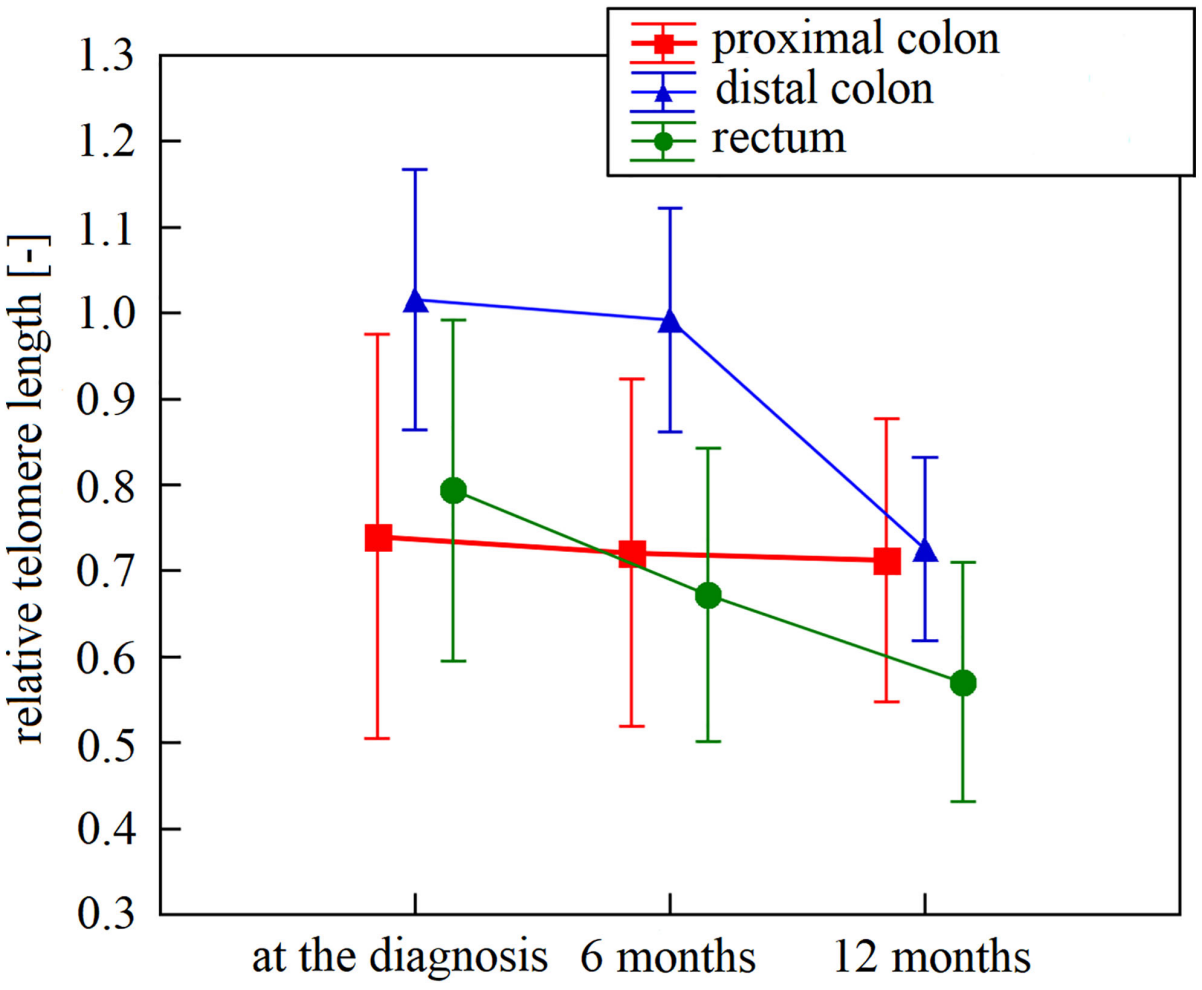
Leukocyte telomere length within 12 months after diagnosis in patients stratified according to their tumor localization. Leukocyte telomere length curve had a different trajectory in patients diagnosed with proximal colon (n = 11), distal colon (n = 24), and rectal cancer (n = 14; p = 0.03). Vertical bars denote 0.95% CI. N represents the number of patients for whom all intervals were available.

The first three samplings (collected at diagnosis and 6 and 12 months after diagnosis) were available for 49 patients. Specifically, we monitored the overall LTL dynamics and dynamics across the patient subgroups. Monitoring of these 49 patients identified LTL shortening during the treatment and follow-up examinations. In the whole group, there was a significant downward trend across the first three 6-month observation periods independent of age, cancer stage, tumor localization, or treatment (repeated measures ANOVA, F(2, 96) = 16.93, n = 49, p < 0.0001). The Bonferroni *post hoc* test revealed that the most pronounced decrease in LTL was between 6 and 12 months after the diagnosis (Bonferroni corrected p < 0.0001, **Figure 1**). Furthermore, patients analyzed in the subgroups categorized according to their tumor location showed different trajectories of LTL decreases. (repeated measures ANOVA F(4, 90) = 2.83, p = 0.03, proximal colon n = 11, distal colon n = 24, rectum n = 14, **Figure 2**). Subsequent analysis with a linear mixed-effects model confirmed the downward time trend of LTL also in the whole patient cohort (average decrease of 1.05% of the initial value per month with a 95% CI of 0.64–1.47%, p < 0.0001), independent of TNM stage, primary tumor location, radiotherapy, or 5-fluorouracil application. The dynamics of LTL in time can also be seen in the differences in LTL values between the measured time points calculated for each patient and summarized in **Table 2**. Concerning therapy response and cancer staging, we did not observe a difference in the rate of LTL decrease between good (n = 19) and poor responders (n = 4) (repeated-measures ANOVA, F(2, 42) = 1.30, p = 0.28), or between TNM I + II (n = 33) and III + IV (n = 13) (repeated-measures ANOVA, F(2, 88) = 0.52, p = 0.60). We also performed sampling 18 months after the diagnosis, but the four complete samples were drawn from 6 patients only.

**Table 2:** Differences in leukocyte telomere length within 1 year from diagnosis.

Time interval						
	n	mean (95% CI)	median	lower quartile	upper quartile	range (min – max)
**LTL 6 months** – **LTL at the diagnosis**	96	-0.031 (-0.083 – 0.021)	-0.023	-0.119	0.092	-0.833 – 0.715
**LTL 12 months** – **LTL 6 months**	97	-0.101 (-0.158 – -0.044)	-0.085	-0.204	0.066	-0.938 – 0.788
**LTL 12 months** – **LTL at the diagnosis**	87	-0.164 (-0.220 – -0.109)	-0.121	-0.368	0.020	-0.957 – 0.388

The table compares delta LTL values between time point intervals.

### 3.2 Leukocyte telomere length in patients prior to therapy vs. clinicopathological data

We observed no correlation between LTL and age at diagnosis (Kendall’s τ = 0.03, p = 0.63, n = 137), gender (females: 0.86 ± 0.35, n = 51; males: 0.86 ± 0.35, n = 86; two-sample *t*-test t(135) = -0.01, p = 0.99), or smoking status (smokers: 0.88 ± 0.34, n = 32; non-smokers: 0.87 ± 0.35, n = 98; two-sample *t*-test t(128) = -0.13, p = 0.90). There was no difference in LTL between patients with microsatellite stability (0.86 ± 0.34, n = 94) and instability (0.82 ± 0.29, n = 22; two-sample *t*-test t(114) = 0.48, p = 0.63). Patients diagnosed with TNM stage I + II (0.84 ± 0.35, n = 82) and TNM stage III + IV (0.89 ± 0.34, n = 47) did not show statistically significant differences in LTL (two-sample t-test t(127) = -0.693, p = 0.49). Likewise, there was no difference in LTL in patients with tumors in the proximal colon, distal colon, and rectal locations (one-way ANOVA (F(2, 132) = 0.28, p = 0.76). The complete LTL results are listed in **Table 3**.

**Table 3:** Leukocyte telomere length in colorectal cancer patient subgroups at the time of the diagnosis.

Clinicopathological characteristics of patients				
		n	leukocyte telomere length mean ± SD	p value
**Sampling collected at the time of the diagnosis**		137	0.86 ± 0.35	-
**Gender**				
	males	86	0.86 ± 0.35	0.99
	females	51	0.86 ± 0.35	
**Smoking status**				
	smokers	32	0.88 ± 0.34	0.90
	non-smokers	98	0.87 ± 0.35	
**Tumor site**				
	proximal colon	31	0.83 ± 0.36	0.76
	distal colon	69	0.87 ± 0.37	
	rectum	36	0.83 ± 0.31	
**UICC TNM stage**				
	I + II	82	0.84 **±** 0.35	0.49
	III + IV	47	0.89 **±** 0:34	
**Microsatellite status**				
	stable	94	0.86 **±** 0.34	0.63
	instable	22	0.82 **±** 0.29	

The table summarises all the results of LTL and stratifies the studied group of CRC patients based on the clinicopathological characteristics.

### 3.3 Linking leukocyte telomere length to treatment response and therapy effect

**Figure 3:**
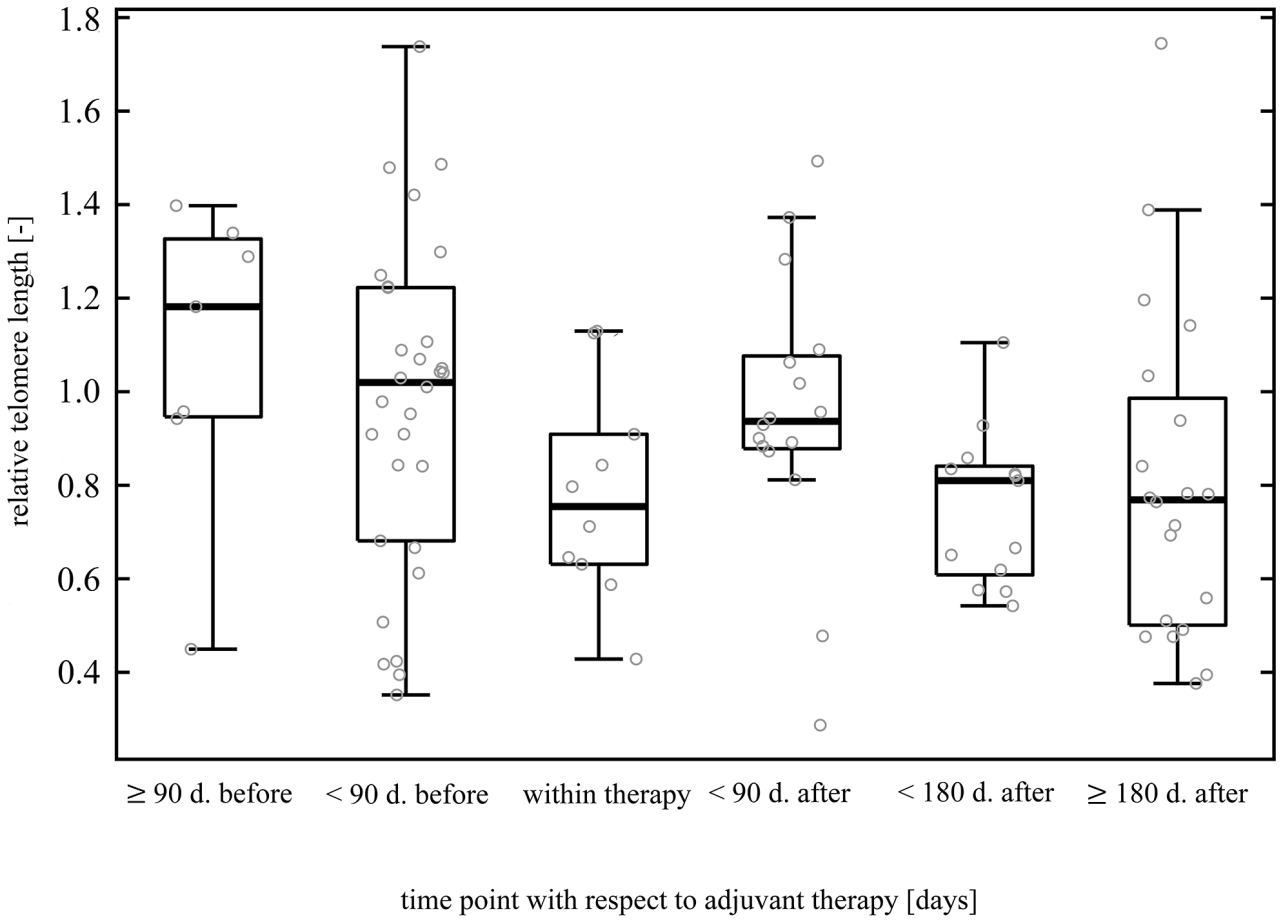
Dynamics of leukocyte telomere length before, during, and post adjuvant treatment. The data originate from patients subjected to surgery followed by adjuvant therapy. Patients with pre-operative treatment were excluded to filter out a possible neoadjuvant therapy influence. The figure comprises 96 data points (samplings) without regard to patients. Forty-two patients redounded to the figure by at least one sampling (3 patients by one sampling, 25 patients by two samplings, 13 patients by three samplings, and 1 patient by four samplings). LTL was significantly non-uniform among the time intervals (p = 0.03), but multiple comparison test did not find partcular differences between any pair of time points.

In incident CRC cases, comparison of LTL between good (0.94 ± 0.34, n = 41) and poor (0.91 ± 0.30, n = 20) therapy responders revealed no difference (two-sample t-test t(59) = 0.34, p = 0.74). With regard to adjuvant therapy, LTL was inversely correlated with the time elapsed from the therapy completion (Kendall’s τ = -0.22, p = 0.03), but the correlation was computed only by using n = 49 data points. Further, LTL was significantly non-uniform across the time intervals defined with respect to the treatment (Kruskal-Wallis ANOVA H(5, 96) = 12.74, p = 0.03, **Figure 3**), although the *post hoc* test did not confirm the differences between any of the time point pairs. Additionally, within the period < 90 days after the end of adjuvant therapy, we found that increase in LTL showed a borderline association with an increased overall survival (Cox proportional-hazards model, hazard ratio (HR) = 0.01, 95% confidence interval (CI) 0.00–0.01, p = 0.05, n = 16). However, stratification based on median LTL (cut-off value 0.94), in contrast, did not show that LTL positively affected the overall survival of the patients (Gehan-Wilcoxon test, p = 0.06). When focusing on neoadjuvant therapy in rectal cancer cases only, LTL of the patients measured before (0.73 ± 0.28, n = 7) and after the therapy administration (0.82 ± 0.36, n = 32) did not differ (Mann-Whitney U = 94, p = 0.53). We also did not find any correlation with time after neoadjuvant therapy (Kendall’s τ = -0.06, p = 0.65). The results are summarized in **Table 4**.

**Table 4:** Leukocyte telomere length with respect to treatment response and therapy effect.

Stratification of the patients based on their therapy				
		n (subjects)	leukocyte telomere length mean ± SD	p value
				-
**Overall therapy response in CRC patients**				
	good	41	0.92 ± 0.34 (at the diagnosis)	0.74
	poor	20	0.90 ± 0.30 (at the diagnosis)	
**Rectal cancer patients with neoadjuvant therapy**				
	pre-	7	0.73 ± 0.28 (before the therapy)	0.53
	post-	32	0.82 ± 0.36 (after the therapy)	
		n (time points)	Kendall rank correlation	p value
**LTL vs. time after adjuvant therapy**		49	-0.22	0.03
**LTL vs. time after neoadjuvant therapy**		32	-0.06	0.65

The table shows analyses on telomere length changes related to therapy.

### 3.4 Leukocyte telomere length as a predictor of overall survival rate

The 3-year overall survival rate of CRC patients was 83.3% (95% CI 76.2–90.4%). Investigating overall survival according to telomere length measured in newly diagnosed patients suggested that LTL had no relation to the survival rate (Cox proportional-hazards model, HR = 1.36, 95% CI 0.33–5.59, p = 0.67, n = 137).

### 3.5 TERT expression in incident cases vs. clinicopathological data

We also measured *TERT* expression in mononuclear cells at the time of diagnosis. *TERT* expression did not correlate with LTL (Kendall’s τ = 0.00, p = 1, n = 104). *TERT* expression was higher in females (n = 43) than males (n = 66) (two-sample *t*-test t(107) = -3.01, 1.35-fold, 95% CI 1.11–1.65, p = 0.003, **Supplementary Figure 3**) and smokers (n = 29) than non-smokers (n = 74) (two-sample *t*-test t(101) = -2.11, 1.27-fold, 95% CI 1.01–1.61, p = 0.04, **Supplementary Figure 4**). No correlation was observed between *TERT* expression and patient age (Kendall’s τ = -0.08, p = 0.23, n = 109). Microsatellite unstable patients (n = 15) showed only negligibly lower *TERT* expression than microsatellite stable patients (n = 75) (0.91-fold, 95% CI 0.67–1.23, two-sample t-test t(88) = 0.66, p = 0.51), as did TNM III + IV (n = 37) in comparison with TNM I + II (n = 66) (0.96-fold, 95% CI 0.79–1.17, two-sample t-test t(101) = 0.37, p = 0.72). When compared to patients with the tumors in the distal colon (n = 53), decrease in TERT expression was observed for the localization in proximal colon (n = 24, 1.12-fold, 95% CI 0.85–1.47) as well as in rectum (n = 31, 1.11-fold, 95% CI 0.86–1.44), however without statistically significant (one-way ANOVA F(2, 105) = 0.55, p = 0.58). Similarly, *TERT* was insignificantly decreased in poor therapy responders (n = 18) compared to good responders (0.87-fold, 95% CI 0.65–1.15, two-sample t-test t(45) = 0.96, p = 0.34).

## 4 Discussion

*In vitro* studies have provided evidence that conventional chemotherapies may alter telomere homeostasis through specific mechanisms of action (5). Thus, there is a biological plausibility that anticancer treatment may be a possible reason for permanent or transient telomere length changes, and these changes may reflect cancer patients’ outcomes. Furthermore, telomere length presumably varies depending on the therapy regimen (7).

To our knowledge, only one longitudinal study has evaluated LTL before and after exposure to chemotherapy in CRC patients (10). In this study, LTL was shortened after standard-dose combination chemotherapy, and the degree of shortening corresponded to good therapy response and neutropenia severity. However, the study also included patients with other solid cancers (n = 29), and the number of patients with CRC was undersized (n = 3). Additionally, Garg et al. found that in CRC patients, a short LTL might predict mucositis as a side effect of 5-FU in the adjuvant setting (23).

In this study, we observed that CRC therapy exerted a moderate effect on LTL, particularly in association with adjuvant therapy. We recorded the LTL correlation with time elapsed after adjuvant therapy, but we did not observe any LTL differences between patients with and without neoadjuvant therapy or between good and poor therapy responders. However, in a recent study on rectal cancer patients, Rampazzo et al. investigated eight SNPs in *TERT*, from which several differentially contributed to LTL erosion during neoadjuvant therapy (24). Furthermore, low levels (≤ median value) of circulating TERT mRNA in plasma and its stable/decreasing levels after neoadjuvant therapy were independently associated with a better therapy response compared to the levels measured before the start of the treatment (24). The assessment of the telomere homeostasis with the treatment response among CRC patients must be conducted after a stringent control for phenotypic tumor heterogeneity and a large variety of treatment regimens. For instance, our previous study showed significant differences in telomere length measured in CRC tumors and adjacent mucosa, related to localization, microsatellite instability status, and stage (20).

Of the limited number of studies investigating the effect of chemotherapy on telomere homeostasis in solid tumors, only two have suggested post-treatment LTL recovery (7). As Benitez-Buelga et al. reported that the recovery phase began approximately two years after diagnosis (7), there is an indication of epidemiological evidence regarding this phenomenon in solid tumors. However, the results of studies on leukemia patients documented the post-treatment telomere recovery phase (5). Our study did not find any normalization of LTL after treatment in CRC patients monitored for approximately 12 months after diagnosis. Longer observations could not be obtained owing to the lack of samples collected 18 months after diagnosis.

In concordance with our results, influence of tobacco smoking and sex on telomerase activity in peripheral blood mononuclear cells from healthy subjects was reported in a previous study, with higher telomerase activity in women and smokers (25). This study also showed that the CpG sites in the *TERT* promoter are differentially methylated in smokers, which suggests that smoke-related oxidative stress has a possible epigenetic effect on *TERT*. In their study, smokers displayed longer telomeres than non-smokers in addition to increased telomerase activity. Telomerase activity/*TERT* expression upregulation in females can be explained by estrogens stimulating telomerase expression (26).

Overall, the main novelty of our work is that, as the first one, we investigated the effect of chemotherapy on LTL solely in patients with CRC. The study is unique because of its follow-up design, which enabled monitoring of LTL from patient diagnosis through treatment and follow-up intervals. However, the number of patients sampled every six months during the follow-up visits declined steadily. After the surgical procedure, patients most likely did not always attend the hospital regularly as planned; blood samples in some cases remained uncollected, and some patients moved to receive follow-up care at their place of residence or even died. We are aware that the majority of repeated samples, therefore, originate from patients with good therapeutic responses.

## 5 Conclusion

This study focused on mapping LTL in patients with CRC since their diagnosis, receiving treatment, and during follow-up care. Our research has demonstrated that LTL decreased 6 months after the approximate end of the therapy (i.e., 12 months from the diagnosis). Moreover, these LTL changes probably have different dynamics, depending on the anatomical localization of the tumors. Leukocytes also likely reflect the effect of adjuvant treatment, as we observed a correlation between LTL shortening and termination of adjuvant therapy. A high variance in treatment regimens suggests that it would be worthwhile to delve deeper into particular treatment strategies and study their possible effects separately. The higher *TERT* expression in women and smokers likely reflects the impact of estrogen hormones and tobacco smoking, respectively. Thus, estrogen- and smoke-induced oxidative stress may induce higher telomerase activity in peripheral blood mononuclear cells.

## Data availability statement

The datasets presented in this study can be found in online repositories. The names of the repository/repositories and accession number(s) can be found in the article/**Supplementary Material**.

## Ethics statement

The studies involving human participants were reviewed and approved by ethics committees of the Institute of Experimental Medicine, The Czech Academy od Sciences, and the Institute for Clinical and Experimental Medicine and Thomayer Hospital. The patients/participants provided their written informed consent to participate in this study.

## Author contributions

KT: data curation (equal), funding acquisition (supporting), investigation (lead), vizualisation (equal), writing original draft (lead), writing review, and editing (equal). MKr: data curation (equal), investigation (supporting), methodology (equal), vizualisation (equal), writing review, and editing (equal). AZ: methodology (equal), writing review, and editing (equal). MKo: supervision (equal). VV: conceptualization (equal), writing review, and editing (equal). PS: resources (equal). LS: resources (equal). ML: resources (equal). KH: supervision (equal). VL: supervision (equal), PH: formal analysis (lead), writing review, and editing (equal). RK: supervision (equal), writing review, and editing (equal). LV: project administration (equal). PV: conceptualization (lead), funding acquisition (lead), project administration (equal), supervision (lead), writing review, and editing (equal). All authors contributed to the article and approved the submitted version.

## Funding

We are grateful for the financial support from the Grant Agency of the Czech Republic (GACR 19-10543S, GACR 21-27902S), the Ministry of Health of the Czech Republic (AZV NV 18-03-00199 and AZV NU 21-03-00145), and the Grant Agency of the Charles University (project GA UK No. 120, UNCE/MED/006). This publication is part of a project that has received funding from the European Union’s Horizon 2020 research and innovation program under grant agreement No. 856620. This study wassupported by project No. CZ.02.1.01/0.0/0.0/16_019/0000787 ‘Fighting INfectious Diseases’ awarded by the MEYS CR.”, financed from EFRR.

## Acknowledgments

We wish to thank the patients for their participation in the study.

## Conflict of interest

The authors declare that the research was conducted in the absence of any commercial or financial relationships that could be construed as a potential conflict of interest.

## Publisher’s note

All claims expressed in this article are solely those of the authors and do not necessarily represent those of their affiliated organizations, or those of the publisher, the editors and the reviewers. Any product that may be evaluated in this article, or claim that may be made by its manufacturer, is not guaranteed or endorsed by the publisher.
