# Supplementary material for: Monitoring of telomere dynamics in peripheral blood leukocytes in relation to colorectal cancer patients’ outcomes
Source: Front Oncol. 2022 Sep 20;12:962929. doi: 10.3389/fonc.2022.962929 (PMC9530927; doi:10.3389/fonc.2022.962929)
Supplement: Supplementary file 1 [file DataSheet_1.docx]

Supplementary Material

## Supplementary Figures


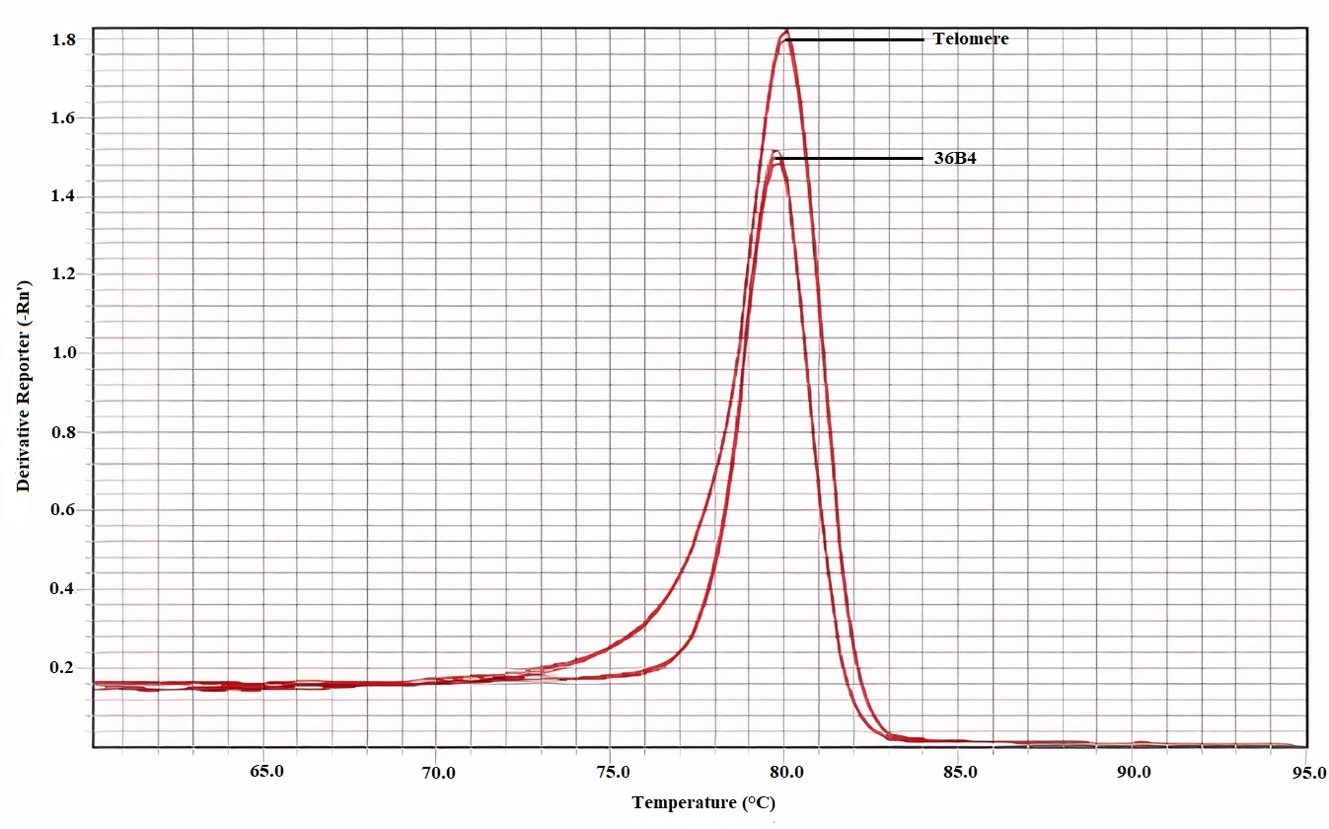


**Supplementary Figure 1. Melting curve for telomere and for the single-copy gene *36B4*.** Both amplified products show the same melting temperature and single peaks indicating specific amplification. By default, the melt assay was set at 95 °C for 15 sec, 60 °C for 1 min, followed with the continuous signal acquisition to 95 °C (ramp rate 0.05 °C/sec) maintained for 15 sec.


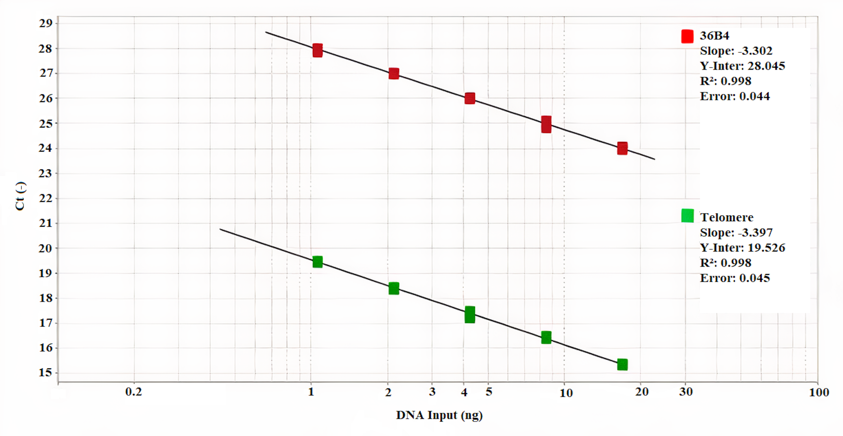


**Supplementary Figure 2. Standard curve for telomere and for the single-copy gene *36B4*.** Standard curves are displayed using a linear regression model. In particular assays, R^2^ ≥ 0.990 was required. The slope of the regression line close to -3.320 is optimal and assumes the reaction efficiencies ≈ 100%.


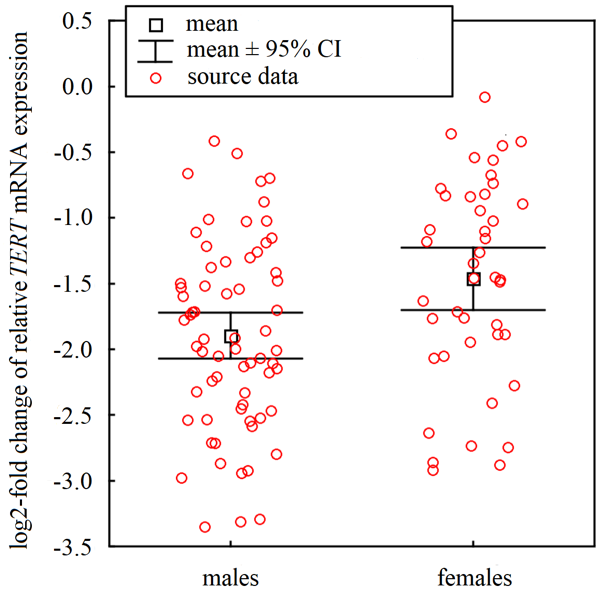


**Supplementary Figure 3. Sex-related log2-transformed differential expression of the gene *TERT*.** Females (n = 43) were shown to have higher *TERT* expression profile compared to males (n = 66) (1.35-fold, 95% CI 1.11–1.65, p = 0.003).


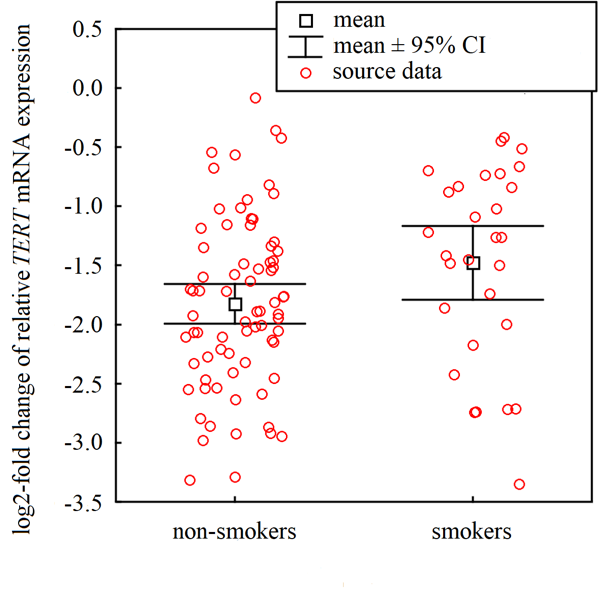


**Supplementary Figure 4. Log2-transformed *TERT* expression changes based upon cigarette smoke exposure.** Smokers (n = 29) were found to have higher *TERT* expression compared to non-smokers (n = 74) (1.27-fold, 95% CI 1.01–1.61, p = 0.04).
