# Supplementary material for: Monitoring of telomere dynamics in peripheral blood leukocytes in relation to colorectal cancer patients’ outcomes
Source: Front Oncol. 2022 Sep 20;12:962929. doi: 10.3389/fonc.2022.962929 (PMC9530927; doi:10.3389/fonc.2022.962929)
Supplement: Supplementary file 2 [file Table_1.pdf]

Supplementary Table 1: Therapy regimens for patients participating in the study. Patients were treted in Thomayer University Hospital in Prague (except where stated otherwise). As to adjuvant therapy, combination of chemotherapy drugs prescribed were: FOLFOX (folinic acid, 5-fluorouracil, and oxaliplatin), FUFA (folinic acid, 5-fluorouracil), FOLFIRI (folinic acid, 5-fluorouracil, irinotecan), XELOX (a prodrug of 5-fluorouracil, oxaliplatin), XELIRI (a prodrug of 5-fluorouracil, irinotecan), and TAS-102 (trifluridine-tipiracil hydrochloride mixture). Other chemotherapeutic agents prescribed were: XELODA (Capecitabine - a prodrug of 5-fluorouracil), Avastin (Bevacizumab - vascular endothelial growth factor inhibitor), Vectibix (Panitumumab - vascular endothelial growth factor inhibitor), Erbitux (Cetuximab - vascular endothelial growth factor inhibitor), Zaltrap (Aflibercept - vascular endothelial growth factor inhibitor). As to neoadjuvant therapy, patients received the same chemotherapy regimens described above, combinations of chemotherapy and radiotherapy, or radiotherapy alone. Gy [Gray] is a derived unit of radiation dose, eV [electronvolt] is a unit of energy. Linear accelerators for radiotherapy typically generate an electron beams with energy of 4-20 MeV. NDA stands for No Data Available.

PATIENT	THERAPY									LEUKOCYTE TELOMERE LENGTH						
**ID**	**Neoadjuvant therapy** (1=yes, 0=no)	**Neoadjuvant therapy** (from date)	**Neoadjuvant therapy** (till date)	**Neoadjuvant therapy regimen**	**Adjuvant therapy** (1=yes, 0=no)	**Adjuvant therapy** (from date)	**Adjuvant therapy** (till date)	**Adjuvant therapy regimen**	**Therapy response** (good=0, poor=1)	**At diagnosi**	**6 months**	**12 months**	**18 months**	**24 months**	**30 months**	**36 months**
**1**	0				0					1,10	0,81	0,61	0,56			
**2**	1	15.09.2008	15.10.2008	XELODA + radiotherapy (6-MeV linear accelerator, 45Gy)	1	15.01.2009	15.05.2009	2x XELOX, 4x XELODA	0	1,54	0,92		0,92			
**3**	0				0					1,09	0,91					
**4**	0				0				0		0,82	0,83	0,76			
**5**	1	15.09.2008	NDA	2x FOLFOX	1	15.10.2008	15.04.2009	10x FOLFOX	0		0,94	0,91	0,71			
**6**	0				1	15.05.2008	15.09.2008	FUFA	1		0,81		0,84	0,78		
**7**	0				1	September 2008	March 2010	(August 2009 - October 2009), XELODA (December 2009 - March 2010)	1		1,34	1,35	1,43			
**8**	0				1	15.03.2008	15.08.2008	XELODA	1		0,96	1,74				
**9**	0				1	15.01.2009	15.05.2009	June 2009), FOLFIRI+Avastin (December 2009 - March 2010), XELODA (June - August 2010), Irinotecan (weekly since August 2010 - April 2011), FOLFOX retreatment (5/2011 - 7/2011)	1	1,48	0,82	1,20				
**10**	1	12.12.2007	17.01.2008	radiotherapy (6-MeV linear accelerator, low-dose rate 44Gy)	0						0,86		0,75			
**11**	1	NDA	NDA	chemoradiotherapy (FNKV)	0					1,49	1,37	1,18		0,78		
**12**	0				1	15.01.2009	15.06.2009	FOLFOX	0	0,68	0,91	0,65				
**13**	0				1	15.02.2009	15.06.2009	12x FOLFOX	0	0,84		0,86				
**14**	1	15.03.2008	15.05.2008	XELODA + radiotherapy (6-MeV linear accelerator, 46Gy)	0				0		1,38	1,30	1,14			
**15**	1	15.01.2009	15.02.2009	XELODA + radiotherapy (6-MeV linear accelerator, 46Gy)	1	March 2009	15.12.2009	FOLFOX (March 2009 - September 2009) + FOLFIRI (April 2009) + Avastin	1	0,91		1,10				
**16**	0				0						0,66	0,95	0,48			
**17**	0				0					1,37		0,89	0,48			
**18**	0				1	13.01.2011	15.09.2015	Avastin (26.06.2011 - 01.10.2012), 13x Vectibix (18.02.2013 - 12.08.2013), 12x XELODA+TAS-102 (15.01.2015 - 15.09.2015)	0	1,60	1,39	1,06				
**19**	0				0					1,05		1,07				
**20**	1	15.03.2009	15.04.2009	XELODA+radiotherapy (low-dose rate 46Gy)	1	15.03.2010	15.04.2010	2x XELODA	1		0,61	0,60				
**21**	0				0					0,54	0,49	0,34				
**22**	1	15.06.2009	15.08.2009	radiotherapy (6-MeV linear accelerator, 46Gy)	0					0,39	0,29	0,28				
**23**	1	15.06.2009	15.07.2009	XELODA + radiotherapy (6-MeV linear accelerator, 46Gy)	1	15.10.2009	15.02.2010	XELODA, 4x Bleomycin+Etoposide+Cisplatin, 2x Etoposide+Cisplatin	0	0,79	0,73	0,58				
**24**	0				0					0,86		0,54				
**25**	0				1	15.03.2006	01.03.2007	8x FOLFOX, 12x FOLFIRI	1						0,71	0,77
**26**	0				1	15.08.2009	August 2011	(12/2010 - 8/2011)	1	0,51		0,48				
**27**	0				1	24.08.2009	29.01.2010	2x FOLFOX (24.08.2009 - 21.09.2009), 10x FUFA (de Gramont regimen, 21.09.2009 - 29.01.2010)	0	0,61	0,71	0,54		0,39		
**28**	1	15.06.2009	15.09.2009	XELODA+radiotherapy (low-dose rate 45Gy)	1	15.11.2009	15.03.2010	XELODA	0		0,98	0,88	0,53			
**29**	1	NDA	NDA	radiotherapy (unknown dosing, outside of Thomayer Hospital in Prague)	0					0,83	0,80					
**30**	1	15.11.2009	15.12.2009	XELODA + radiotherapy (6-MeV linear accelerator, low-dose rate 46Gy)	0				0	0,97		0,95				
**31**	0				0					0,98		0,75				
**32**	0				1	15.08.2000	15.02.2001	6x FUFA		0,76		1,12				
**33**	1	15.11.2009	15.12.2009	XELODA + radiotherapy (low-dose rate 46Gy)	1	15.11.2009	15.12.2009	6x XELODA	0		1,17	0,99	0,74			
**34**	1	NDA	NDA	radiotherapy (unknown dosing, outside of Thomayer Hospital in Prague)	1	15.11.2009	15.06.2010	Paliative 6x FOLFOX+Avastin			1,13	1,02				
**35**	0				1	15.03.2010	15.09.2010	11x FOLFOX + 12x FUFA	1	1,09		0,69				
**36**	0				0						1,40	1,17	0,89	0,69	0,68	
**37**	1	15.04.2010	NDA	unknown, outside of Thomayer Hospital in Prague	NDA					1,19	1,14					
**38**	0				0					0,82	0,63					
**39**	0				0					0,93	1,08	0,65				

**40**	0				1	18.05.2010	12.10.2010	6x FUFA	0	1,03	1,09					
**41**	0				0					0,57	0,47	0,37				
**42**	1	15.04.2010	15.06.2010	XELODA + radiotherapy (6-MeV linear accelerator, 46Gy)	1	15.08.2010	15.03.2011	XELODA	0	1,08	0,73	0,53				
**43**	0				1	15.05.2010	15.10.2010		0	1,25	0,48					
**44**	0				1	27.05.2010	17.11.2010	12x FOLFOX	1	0,98	0,94	0,58				
**45**	0				1	25.06.2010	06.12.2010	2x XELOX, 6x XELODA	1	0,85		0,59				
**46**	0				1	15.09.2009	NDA	30.08.2010)+Avastin, 8x FOLFIRI (since November 2010), 2x FUFA (since February 2011)	1	0,87	0,94					
**47**	1	NDA	NDA	NDA	0						0,91	1,00	0,94			
**48**	1	15.04.2010	15.05.2010	XELODA+radiotherapy (6-MeV linear accelerator, 46Gy)	0				0		1,09	1,06	1,04			
**49**	1	15.04.2010	15.05.2010	XELODA+radiotherapy (6-MeV linear accelerator, 46Gy)	1	15.08.2010	15.10.2010	3x XELODA	0		0,59	0,52	0,62			
**50**	1	15.03.2010	15.04.2010	XELODA+radiotherapy (6-MeV linear accelerator, 46Gy)	1	15.08.2010	15.12.2010	6x XELODA	0		0,61	0,50	0,35			
**51**	1	15.06.2010	15.07.2010	XELODA+radiotherapy (low-dose rate 46Gy)	1	01.11.2010	08.11.2010	1x XELODA - not completed	0	0,80	0,74	0,57				
**52**	0				0					0,54	0,57					
**53**	0				0						0,45	0,58	0,53			
**54**	0				0					1,03	1,14					
**55**	0				0					0,59	0,59	0,44	0,40			
**56**	0				1	15.10.2010	15.04.2011	6x FOLFOX	1	0,96	0,93					
**57**	1	15.08.2010	15.09.2010	XELODA+radiotherapy (6-MeV linear accelerator, low-dose rate 45Gy)	0				1	0,76	0,81					
**58**	1	18.08.2010	24.09.2010	XELODA+radiotherapy (low-dose rate 45Gy/25fr)	0				0	0,67	0,51	0,40				
**59**	0				0					0,73	0,90					
**60**	1	15.09.2010	15.10.2010	XELODA+radiotherapy (45Gy)	1	15.12.2010	15.04.2011	3x XELODA, 2x FUFA	0	1,21	0,60	0,69	0,58			
**61**	0				0					0,79	0,84					
**62**	0				1	13.09.2010	26.09.2010	1x XELOX	1	0,67	0,57					
**63**	1	27.09.2010	22.11.2010	3x XELOX	1	21.03.2011	14.09.2012	XELIRI+Avastin (21.02.2012 - 07.08.2012), 2x XELODA+Avastin (5.08.2012 - 14.09.2012)	1	0,72	0,76					
**64**	0				0					0,86	0,39		0,44			
**65**	1	15.09.2010	15.10.2010	XELODA+radiotherapy (6-MeV linear accelerator, 45Gy)	1	15.01.2011	15.05.2011	6x XELODA	0	0,68	0,74	0,40				
**66**	1	15.06.2010	15.07.2010	radiotherapy (6-MeV linear accelerator, low-dose rate 46Gy)	0				0		1,20	0,79				
**67**	0				1	10.11.2010	23.04.2011	12x FOLFOX+Avastin (since 3. cycle)	0	1,22	0,80	0,93				
**68**	0				0					0,96	0,73	0,75				
**69**	0				1	15.11.2010	15.04.2011	XELODA	0	0,95	0,90	0,67				
**70**	1	15.11.2010	15.05.2011	palliative chemotherapy - 12x FOLFOX + Avastin	1	15.08.2011	15.02.2013	FOLFIRI, FUFA, Avastin, XELODA	1	1,10	1,27					
**71**	0				1	15.09.2011	15.02.2012	FUFA	1	0,94	1,18					
**72**	0				1	16.12.2010	09.05.2011	6x FUFA	0	1,04	1,06	1,39				
**73**	0				0					1,25		0,84				
**74**	1	15.04.2010	15.11.2010	palliative chemotherapy - 12x FOLFOX+Avastin	1	15.04.2011	15.11.2011	10x FOLFIRI	0		0,81	0,73				
**75**	0				1	15.12.2011	15.05.2012	XELODA	0	1,34	0,84					
**76**	0				1	01.11.2012	01.03.2013	FUFA	1	1,29		0,81				
**77**	0				1	29.06.2012	01.11.2012	FUFA	0	0,84		0,48	0,38			
**78**	0				0					0,98	1,06					
**79**	1	15.09.2012	05.11.2012	XELODA+radiotherapy (6-MeV linear accelerator, 40Gy)	1	01.12.2012	01.06.2013	XELOX, XELODA	0		1,01	0,89				
**80**	1	15.09.2011	15.10.2011	radiochemotherapy (unknown dosing, outside of Thomayer Hospital in Prague)	0						0,94	1,11				
**81**	0				0					1,36	1,37	0,87				
**82**	0				0					1,76		1,72				
**83**	1	29.08.2012	11.10.2012	XELODA+radiotherapy (6-MeV linear accelerator, 44Gy)	1	24.01.2013	24.06.2013	XELODA	0		0,81	0,44	0,55			
**84**	0				0					1,68	1,95					
**85**	0				1	18.01.2013	15.05.2013	XELODA	0	1,74	1,49	0,78				
**86**	1	06.08.2012	15.09.2012	12x FOLFOX	0						1,21		0,66			
**87**	1	15.02.2011	15.03.2011	XELODA+radiotherapy (6-MeV linear accelerator, 44Gy)	1	12.07.2013	01.02.2014	XELODA, FUFA (de Gramont regimen), FOLFOX+Erbitux, FOLFOX, Zaltrap	1	1,16	1,04	0,60				
**88**	1	05.12.2012	14.01.2013	XELODA+radiotherapy (6-MeV linear accelerator, 44Gy)	1	03.04.2013	02.07.2013	XELODA	0		1,53	1,48				
**89**	1	01.10.2012	01.01.2013	palliative chemotherapy - 7x FOLFOX+Avastin	1	11.03.2013	16.06.2013	FOLFOX+Avastin	1		1,81	1,77				

**90**	1	NDA	15.12.2012	XELODA+radiotherapy (50Gy)	1	15.05.2013	October 2013	FOLFOX	0		1,14	0,61	0,67			
**91**	1	NDA	06.12.2012	radiochemotherapy (unknown dosing, outside of Thomayer Hospital in Prague)	0						0,87		0,39			
**92**	0				1	13.03.2013	21.08.2013	FOLFOX+Avastin	1	1,49	1,37	0,62				
**93**	0				1	15.03.2013	15.08.2013	XELODA	0	1,11		0,51				
**94**	1	17.12.2013	28.01.2013	XELODA+radiotherapy (6-MeV linear accelerator, 44Gy)	0						1,00	0,51	0,58			
**95**	0				1	13.05.2013	07.10.2013	6x XELOX, 2x XELODA+radiotherapy	0	1,42	0,59	1,11				
**96**	0				1	29.04.2013	23.07.2013	FUFA	0	1,30				1,14		
**97**	0				0				0	0,76		0,76				
**98**	1	14.01.2012	14.02.2013	XELODA+radiotherapy (6-MeV linear accelerator, 44Gy)	1	25.07.2013	07.10.2013	FUFA, FOLFOX+Vectibix	1		1,24	1,05	0,52			
**99**	1	08.01.2013	07.02.2013	radiotherapy (6-MeV linear accelerator, 44Gy)	0						1,39	1,36	0,77			
**100**	0				NDA					0,91		0,47				
**101**	0				0					0,38	0,49					
**102**	0				0					1,13		0,70				
**103**	0				0					0,51	1,23					
**104**	0				0					1,44	1,65					
**105**	0				0					1,08	1,04	0,63	1,00			
**106**	1	31.07.2013	29.08.2013	XELODA+radiotherapy (6-MeV linear accelerator, 44Gy)	1	22.11.2013	21.03.2014	6x XELOX	0	1,01	1,02	0,61				
**107**	0				1	30.07.2013	11.04.2014	FOLFOX	1	0,91	0,65					
**108**	0				1	26.08.2013	29.01.2014	12x FUFA (de Gramont regimen)	0	1,22	1,28	0,82				
**109**	0				0					1,19	1,76	0,82				
**110**	0				0					1,14	1,10					
**111**	1	15.05.2013	15.06.2013	XELODA+radiotherapy (6-MeV linear accelerator, 44Gy)	1	15.11.2013	15.03.2014	6x FUFA	0		1,25	1,30	1,42			
**112**	0				1	08.11.2014	26.02.2015	3x FUFA (Mayo regimen), 1x XELODA	0	1,21	1,40	1,05				
**113**	0				0					1,03	1,07	1,12				
**114**	0				0					1,34	1,17					
**115**	0				1	07.01.2014	11.06.2014	12x FUFA (de Gramont regimen)	0	1,04	1,13	1,03				
**116**	0				0						1,35	1,25				
**117**	1	14.10.2013	13.11.2013	XELODA+radiotherapy (6-MeV linear accelerator, 42Gy)	1	03.05.2014	28.08.2014	6x XELODA	0		1,38	1,46				
**118**	0				0					0,74	0,77					
**119**	1	15-10-2013	15.11.2013	radiochemotherapy (unknown dosing, outside of Thomayer Hospital in Prague)	1	01.02.2014	15.07.2014	XELOX	0		0,92			0,45		
**120**	0				1	03.03.2014	14.07.2014	5x XELOX, 1x XELODA	0	1,01	0,89					
**121**	0				0						0,64	0,54				
**122**	0				0						0,90	0,96				
**123**	0				0						0,83	0,79				
**124**	0				0					0,95		1,01				
**125**	0				0						0,45	0,56				
**126**	0				0					1,11	0,94	0,95				
**127**	0				1	NDA	NDA	NDA	1	0,71		0,65	0,74	0,31		
**128**	0				0					0,65		0,53				
**129**	0				0					0,96				0,99		
**130**	0				1	24.06.2014	26.11.2014	12x FUFA (de Gramont regimen)	0	0,91		0,83				
**131**	0				1	NDA	15.02.2015	NDA	0	0,93	0,88					
**132**	0				0					0,78	0,79					
**133**	0				0					1,05	1,17	1,05				
**134**	0				0					0,76	0,76	0,73				
**135**	1	02.07.2014	06.08.2014	XELODA+radiotherapy (6-MeV linear accelerator, 44Gy)	1	08.10.2014	23.10.2014	2x FOLFOX	0		0,91	1,09	1,01			
**136**	0				0						0,39	0,88				
**137**	0				0					1,18		1,09				
**138**	1	15.10.2014	25.05.2015	11x FOLFOX, 3x FUFA (de Gramont regimen)	0						1,02	1,07				
**139**	0				0					0,91	0,88	0,39				
**140**	0				0						0,38	0,47				
**141**	0				1	25.03.2015	02.09.2015	2x XELOX (25.03.2015 - 15.04.2015), 8x FUFA (de Gramont regimen, 26.05.2015 - 02.09.2015)	0	1,07	0,93					
**142**	0				0					0,47		0,56				
**143**	0				0					0,41	0,94	0,80				
**144**	1	25.02.2015	27.03.2015	XELODA+radiotherapy (6-MeV linear accelerator, 44Gy)	0						0,72	0,60	0,51			
**145**	0				0				0	1,01	1,38					

**146**	1	01.12.2014	02.02.2015	radiotherapy pelvis minor 45 Gy (+ boost rectum up to 50 Gy)	1	01.03.2015	01.06.2015	3x XELOX			0,46	1,06	0,47			
**147**	1	09.12.2014	14.01.2015	XELODA+radiotherapy (6-MeV linear accelerator, 44Gy)	1	19.05.2015	25.09.2015	FUFA			1,46	0,66	0,82			
**148**	0				1	15.04.2015	04.11.2015	6x XELODA		0,46	0,41	0,48				
**149**	0				1	18.08.2015	30.12.2015	FOLFIRI+Avastin	1	0,44	0,83					
**150**	0				0					0,28	0,52	0,59				
**151**	1				0						0,67		0,83			
**152**	0				0					0,44	0,42	0,50				
**153**	0				0					0,43	0,40	0,48				
**154**	1	28.04.2015	01.06.2015	XELODA+radiotherapy (6-MeV linear accelerator, 44Gy)	1	11.08.2015	02.12.2015	1x XELOX, 3x FOLFOX, 3x FUFA (de Gramont regimen)	0		0,60	0,43	0,35			
**155**	0				0						0,44	0,65				
**156**	1	15.04.2015	18.05.2015	radiotherapy (6-MeV linear accelerator, 44Gy)	0						0,46		0,53			
**157**	0				1	11.09.2015	17.02.2015	12x FOLFOX	0	0,74						0,49
**158**	0				1	19.10.2015	28.06.2016	FOLFOX	0	0,40		0,51	0,38			
**159**	0				0					0,97	0,38			0,41		
**160**	0				0					0,27	0,29	0,34				
**161**	0				0					0,58	0,50					
**162**	0				0				0	0,49	0,54	0,50	0,55			
**163**	1	15.01.2015	03.07.2015	FOLFOX+Avastin	NDA						1,05	0,50	0,65			
**164**	1	16.06.2015	16.07.2015	XELODA+radiotherapy (6-MeV linear accelerator, 44Gy)	1	01.12.2015	01.04.2016	5x FOLFOX, 4x FUFA (de Gramont regimen)	0		0,49	0,55				
**165**	0				0					0,34	0,40					
**166**	1	NDA	30.04.2015	NDA	NDA						0,54		0,54			
**167**	1	22.07.2015	20.08.2015	XELODA+radiotherapy (6-MeV linear accelerator, 44Gy)	1	15.11.2015	15.03.2016	5x XELOX	0		0,58	0,69				
**168**	0				NDA					0,40		0,50				
**169**	0				0					0,55		0,50				
**170**	1	24.09.2015	29.10.2015	XELODA+radiotherapy (6-MeV linear accelerator, 44Gy)	1	NDA	NDA	6x XELODA			0,43		0,70			
**171**	1	25.11.2015	06.01.2016	radiotherapy 45Gy (25 x 1,8 Gy) 18 MeV	1	27.02.2016	24.06.2016	6x XELODA	0	0,48	0,44					
**172**	0				0					0,48	0,71	0,51				
**173**	0				0					0,64		0,56				
**174**	0				1	21.12.2015	25.05.2016	12x FUFA (de Gramont regimen)	0	0,39		0,49				
**175**	1	01.09.2015	02.10.2015	XELODA+radiotherapy (18MeV, 41,4Gy)	1	03.02.2016	05.06.2016	6x XELOX	0		0,28		0,47			
**176**	0				1	NDA	NDA	FOLFOX, XELODA		0,47		0,47				
**177**	1	NDA	April 1015	radiochemotherapy (46Gy)	NDA						0,39	0,46	0,68			
**178**	1	NDA	05.10.2015	radiochemotherapy (unknown dosing, outside of Thomayer Hospital in Prague)	0						0,74	0,25				
**179**	0				0					0,48		0,38				
**180**	0				1	01.02.2016	01.08.2016	XELODA)	0	0,42		0,56				
**181**	0				0					1,13		0,76				
**182**	0				1	16.03.2016	17.06.2016	7x FUFA (de Gramont regimen)	1	0,42	0,29					
**183**	1	NDA	20.08.2015	radiochemotherapy (unknown dosing, outside of FTN)	1	NDA	NDA	XELODA			0,46		0,41			
**184**	0				0					0,47	0,64					
**185**	0				0				0	0,39		0,59	0,54			
**186**	0				NDA					0,58		0,61	0,40			
**187**	1	September 2015	December 2016	6x FOLFOX	1	24.06.2016	01.11.2017	regimen)+Avastin (24.06.2016 - 03.12.2016), 3x Lonsurf (till April 2017), Stivarga (May 2017 - November 2017)	1		0,56		0,45			
**188**	0				0					0,38	0,48	0,41	0,47			
**189**	1	10.03.2016	12.04.2016	XELODA+radiotherapy (6-MeV linear accelerator, 44Gy)	0					0,43		0,56				
**190**	1	08.01.2016	08.02.2016	XELODA+radiotherapy (18-MeV, 44Gy)	0						0,36	0,38				
**191**	0				0					0,55	0,44					
**192**	0				1	30.08.2016	19.02.2017	8x XELODA	0	0,45	0,63					
**193**	0				0					0,44	0,54	0,49				
**194**	0				0					0,58	0,63					
**195**	0				0					0,47	0,37					
**196**	0				1	02.09.2016	28.01.2017	FUFA (Mayo regimen)		0,35	0,43					
**197**	0				0					0,37	0,78					
**198**	0				0					0,47		0,42				
