# Supplementary material for: Monitoring of telomere dynamics in peripheral blood leukocytes in relation to colorectal cancer patients’ outcomes
Source: Front Oncol. 2022 Sep 20;12:962929. doi: 10.3389/fonc.2022.962929 (PMC9530927; doi:10.3389/fonc.2022.962929)
Supplement: Supplementary file 3 [file Table_2.pdf]

Supplementary Table 2: Primer sequences used to quantify telomeres. Leukocyte telomere length was calculated relative to 36B4 (acidic ribosomal phosphoprotein P0).

**PRIMERS**				
	**Name**	**Primer**	**Primer sequence (5'-3')**	**PCR product size (bp)**
**Telomere repeats**				
	Tel 1 1b	Forward	CGGTTTGTTTGGGTTTGGGTTTGGGTTTGGGTTTGGGTT	39
	Tel 1 2b	Reverse	GGCTTGCCTTACCCTTACCCTTACCCTTACCCTTACCCT	39
36B4				
	36B4u	Forward	CAGCAAGTGGGAAGGTGTAATCC	23
	36B4d	Reverse	CCCATTCTATCATCAACGGGTACAA	25
